# Variation in occupational exposure associated with musculoskeletal complaints: a cross-sectional study among professional bassists

**DOI:** 10.1007/s00420-017-1264-5

**Published:** 2017-10-20

**Authors:** Kees H. Woldendorp, Anne M. Boonstra, J. Hans Arendzen, Michiel F. Reneman

**Affiliations:** 1 ‘Revalidatie Friesland’ Center for Rehabilitation, PO Box 2, 9244 ZN Beetsterzwaag, The Netherlands; 2 Department of Rehabilitation Medicine, Leiden University Medical Center, Leiden, The Netherlands; 3 Center for Rehabilitation and Department of Rehabilitation, University Medical Center Groningen, University of Groningen, Groningen, The Netherlands

**Keywords:** Pain, Posture, Musicians, Mono-instrumentalism, Multi-instrumentalism

## Abstract

**Background:**

Variation in occupational exposure is assumed to have a protective effect against the development of musculoskeletal complaints (MSC), but this common assumption is not strongly supported by the literature. Among musicians, who have a high prevalence of MSC, many play more than one type of instrument (multi-instrumentalism) for many hours a day. Since multi-instrumentalism implies greater variation in ergonomic load of specific musculoskeletal areas than mono-instrumentalism, musicians are a suitable study population to test whether the above assumption is true.

**Purpose:**

To investigate in a sample of professional bass players whether multi-instrumentalists are less likely to have MSC than mono-instrumentalists.

**Methods:**

Participants were 141 professional and professional student double bassists and bass guitarists. Demographic, MSC and exposure characteristics were collected online with self-constructed and existing questionnaires. Logistic regression analysis was used to test the association between multi- versus mono-instrumentalism and MSC, adjusted for confounders.

**Results:**

The prevalence of having MSC in the neck, back, right shoulder area and both wrist areas did not differ significantly between the two groups. Further analysis revealed that the likelihood of having MSC in the left shoulder area was higher in multi-instrumentalists compared to mono-instrumentalists (Odds ratio 0.30, 95% CI 0.119–0.753, *p* = 0.010).

**Conclusion:**

In this sample of professional bass players, no protective effect of multi-instrumentalism against MSC was found. Multi-instrumentalism was associated with a higher prevalence of MSC in the left shoulder. This result challenges theoretical and clinical assumptions in occupational and pain medicine.

**Electronic supplementary material:**

The online version of this article (doi:10.1007/s00420-017-1264-5) contains supplementary material, which is available to authorized users.

## Introduction

Among professional musicians there is a high life-time prevalence (up to 93%) of musculoskeletal complaints (MSC), mainly chronic pain in the upper body (Kok et al. 2016; Silva and Afreixo 2015). The impact of pain on playing is substantial and frequently leads to problems in daily activities (Wu 2007) or even to premature career termination (Davies and Mangion 2002; Wu 2007). Identifying the underlying factors is crucial in the prevention and treatment of MSC and disability in musicians.

The underlying mechanism of MSC in this population is still being debated (Baadjou et al. 2016; Bongers et al. 2002; Davies and Mangion 2002; Stock 1991; Wu 2007). Multiple bio-psycho-social factors have been identified in the literature as being associated with MSC in the general working population (National Research Council 2001) as well as among musicians (Baadjou et al. 2016; Kok et al. 2016; Silva et al. 2015; Wu 2007). However, causal relationships are difficult to demonstrate, despite the overwhelming amount of literature. This might be due to the multi-causal nature of MSC, to the cross-sectional survey nature of a majority of the research designs (Baadjou et al. 2016; Wu 2007), the diversity of instrumentalists in the study populations (Baadjou et al. 2016) and/or to other difficulties in meeting the criteria for ascertaining causation (Bradford-Hill 1965). Studies focusing on the role of physical causes of MSC have frequently reported the influence of external (workplace-related factors) and internal (individual) occupational issues on chronic pain (National Research Council 2001; Bongers et al. 2002; Ijmker et al. 2011). According to the conceptual load tolerance model of the National Research Council (2001), three domains of external occupational ‘pathways’ can be distinguished: external loads, organizational factors and the social context. Biomechanical loading depends on interactions between internal tolerances and adverse factors, e.g. adaptation to the loading when internal tolerances are exceeded. Biomechanical loading is also affected by individual characteristics, such as anthropometry and other factors mediating the transmission of external loads to internal loads on anatomical structures of the body. The load-tolerance model should be considered as a model embedded in a bio-psycho-social body concept (Marras et al. 2000). Despite these complex relationships, some associations have been identified between physical attributes and external loads, such as force, posture, vibration and temperature (National Research Council 2001). Among these loads, variation in loading (e.g. through variation in posture) was not explicitly mentioned in the NRC’s report. The above-mentioned associations appear to be true also for musicians (Wu 2007).

One example of variation in loading arises from the fact that musicians can play one type of instrument (mono-instrumentalism) or more than one type of instrument (multi-instrumentalism), and the latter is the case for a significant proportion of the bass player population. In view of the greater variation in occupational loads, playing more than one type of instrument has been suggested to have a protective effect against MSC (Ranelli et al. 2011). Others (Wagner 2005; Storm 2006) warned against the playing of different instruments due to the possible introduction of new MSCs. The association between multi-instrumentalism and MSC, however, has not been tested in a study design involving adjustment for potential confounders.

**Fig. 1:**
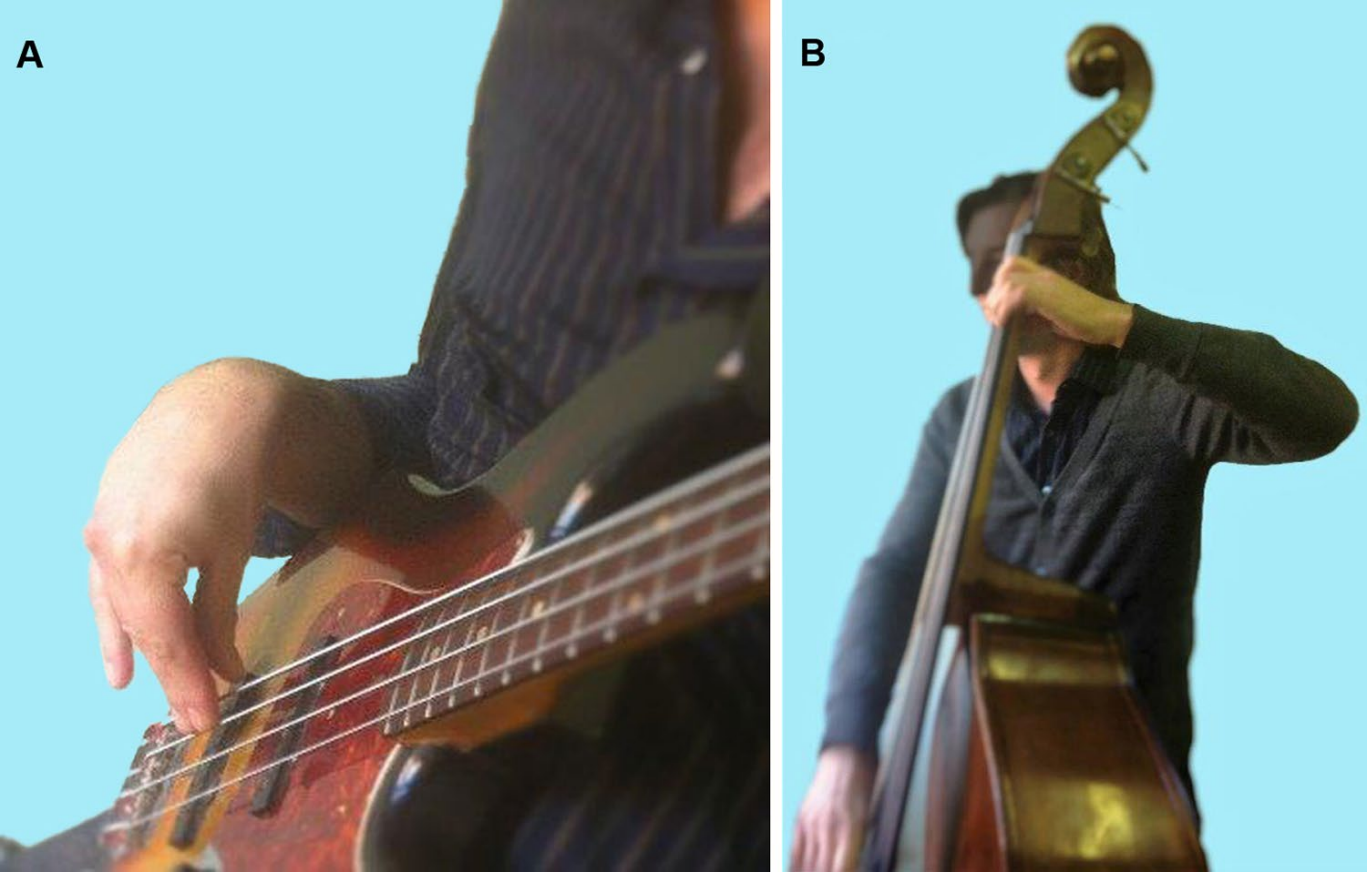
Playing postures of bass guitarists with extreme flexion of the wrist (**a**), and double bassists with abduction of the shoulder/elevation of the upper arm (**b**)

To discover the causal factors for MSC, it is first necessary to find an association. In addition, the mechanism of causation should be theoretically plausible, and the causal factor must be present prior to the onset of the MSC (Bradford Hill 1965). In a previous study, we focused on the first of these aspects, i.e. finding an association between unfavourable occupational load and MSC (Woldendorp et al. 2015). The occupational exposure experienced by musicians varies with the instrument. Bassists are one of the suitable subgroups for studying this association, as there are two types of bass instrument which differ in playing technique and hence in occupational load (the bass guitar and the double bass), and a proportion of bassists play both types and/or another instrument. In addition, playing a double bass or bass guitar involves a ‘poor playing position’ of the left shoulder or right wrist area (see Fig. 1), which increases the risk of MSC in these body parts (‘poor playing position’ is defined as a joint not being held in its mid-range position during playing, and/or a relatively high need for muscle activity against gravity (Woldendorp et al. 2015). This gives us the opportunity to study the impact on MSC of the differences between groups differing in level of variation, focusing on the joint areas of the upper half of the body. Contrary to our assumption, our previous study (with the same study population) found no statistically significant difference between the subgroups of bassists as regards to the prevalence of MSC in the two ergonomically most compromised joint areas: the shoulder area, related to the neck side of the instrument, and the wrist area, related to the box side/bow side of the instrument^1^ (Woldendorp et al. 2015). This would seem to suggest that different postures of playing are not associated with differences in MSC. In the present study, we focused again on finding an association between a causal factor and MSC, in this case on the association between variation in occupational load and the prevalence of MSC in the joint areas (neck, back, shoulders/upper arms and lower arms/wrists) of the upper part of the body.

This study was part of a larger research project about the association between posture and the prevalence of musculoskeletal complaints, conducted in the same sample of professional bassists (Woldendorp et al. 2015). Hence, some parts of the method section are similar to those in our previous paper. In the present study, we tested the null hypothesis that the prevalence of MSC in the joint areas of the upper body among bassists playing at least two types of instruments (multi-instrumentalists) would be similar to that among bassists playing only one type of bass instrument (mono-instrumentalists).

## Methods

### Study design and participants

Participants in this cross-sectional study were professional and conservatory student double bassists and bass guitarists. The bassists were recruited in 2009 and 2013/14 from one Dutch professional orchestra, from three conservatories in the Netherlands and via the website of the International Society of Bassists (www.isbworldoffice.com; Accessed January 2014). Bassists were notified and recruited through their teachers or colleagues and were given further information by a researcher (AT). The group of bassists recruited via the website received information via a standard digital introduction text. Bassists (18 years and older) who were able to fill out the questionnaire in English or Dutch were included if they had graduated from, or were a student, at a conservatory.

All participants provided informed consent. Because of the type of study, involving a questionnaire and healthy volunteers, the Medical Ethics Committee decided that no approval was needed.

### Procedure

All potential participants received a web-based questionnaire, via a URL link. They received the same instructive e-mail and questionnaire, in Dutch or English, depending on their preference. The explanatory information provided to the participants at the start of the study informed them about the need for medical research among musicians, but not about the specific goal of the study.

### Measurements

In the absence of an existing questionnaire, we created a questionnaire suitable for measuring music-related issues among bassists (Online resource 1). Self-reported functioning, height and weight, physical and mental health status, pain location and pain intensity were assessed using questions from existing questionnaires. The combined questionnaire required approximately 20–25 min to complete.

### Mono- and multi-instrumentalism

The bassists were asked whether they played bass guitar, double bass, both bass instruments and/or another instrument for at least 5 h a week each.

Mono/multi-instrumentalism was dichotomized into a score of ‘0’ (mono-instrumentalism) if the bassist played only one type of bass instrument without playing another instrument and ‘1’ (multi-instrumentalism) if they played more than one type of instrument. Bassists were assigned to the ‘multi-instrumentalism’ category if they reported playing for more than 20% of their playing time on their least frequently used bass instrument. They were also assigned to the multi-instrumentalism group if they indicated that they played another instrument for at least 5 h a week. The dichotomization in the case of another instrument was arbitrarily based on exposure to at least 1 h of occupational stress from the instrument nearly every workday. On average, a professional musician plays 1300 h of music a year, i.e. approximately 25 h a week (Paarup et al. 2011); a playing time of at least 5 h a week is, therefore, equivalent to at least one-fifth of the playing time being spent on one other instrument besides their main instrument. Studies (Abréu-Ramos and Micheo 2007; Benjjani et al. 1984; Hochberg and Lederman 1995; Wu 2007) have reported an association between the amount of playing time and the prevalence of MSC, finding that playing an instrument for at least 1 h a day or more had a significant impact.

### Musculoskeletal complaints

The questions regarding MSC were divided into two time-related categories; ‘complaints occurring longer than 3 months ago’ and ‘complaints in the last 3 months’. Respondents ranked each item on a four-point scale ranging from ‘always’, ‘often’, ‘rarely’ to ‘never’. The intensity of pain during the last week was also measured using a Numeric Rating Scale (Hartrick et al. 2003) ranging from ‘no pain’ (score 0) to ‘worst pain’ (score 10). The location of MSC was assessed for the following parts of the upper body half (left or right): neck, back, shoulder, upper arm, elbow, forearm, wrist and/or fingers (see Woldendorp et al. 2015 for the exact definition of the body parts). The analysis was based on the data regarding ‘complaints in the last 3 months’. The pain intensity scores during the last week were used to characterize the population, but not for the analyses, as we assumed that the data from the last week would be too vulnerable to bias due to fluctuations over time.

### Potential confounders

Multiple bio-psycho-social factors have been reported to contribute to chronic pain in musicians (Bragge et al. 2006; de Souza et al. 2012; Pascarelli and Hsu 2001; Wu 2007), and were added to our analysis as potential confounders (for an overview of the potential confounders we studied, see Table 1 and Online Resources 2 and 3).

**Table 1:** Summary of demographic, MSC and exposure characteristics of the bassists playing one type of instrument (mono-instrumentalists), and the bassists playing both bass instruments or one bass instrument and one other instrument (multi-instrumentalists)

	Mono-instrumentalist (*n* = 73)	Multi-instrumentalist (*n* = 68)
Age (yrs; mean (SD))	34.7 (14.2)	35.3 (15.8)
Gender (% male)	86.3	91.2
Sports (% yes)	46.6	50.0
Playing time category (%)		
< 8 h/week	5.5	10.3
≥ 8 h or < 15 h/week	15.1	16.2
≥ 15 h or < 22 h/week	23.3	32.4
≥ 22 h/week	56.2	41.2
Bowing type %		
French bowing	30.1	36.8
German bowing	20.5	27.9
Both	4.1	11.8
No bow or don’t play double bass	45.2	23.6
Playing bass guitar (% yes)	39.7	17.6
Playing double bass (% yes)	60.3	17.6
Playing both (% yes)	0	64.7
Classical music (% yes)	57.5	73.5
Jazz music (% yes)	83.6	98.5
Pop music (% yes)	83.6	91.2

Data on playing characteristics was assessed using items 1.5, 2.7–2.10, 2.12, 2.13, 2.15–2.17, 5.1 and 5.2 of the International Society of Bassists ‘Body and Bass’ Survey (ISBS) (Gilbert 2008). The ISBS is a short, descriptive, non-validated questionnaire which is used to collect reliable information specifically relating to bassists (Gilbert 2008). It includes 42 items divided over 5 dimensions, regarding technical playing aspects, physical symptoms, mental/emotional symptoms, general information and two open questions allowing the opportunity to suggest anything that could diminish complaints.

The prevalent playing positions of the study participants were later researched on the Internet. General health status was assessed with one question of the Short Form 36 Health Survey [item 1 (Stewart and Ware 1992)]; referred to as ‘subjective health score’. Psychological distress was assessed with the Brief Symptom Inventory (BSI) (Derogatis and Melisaratos 1983). The BSI is the shortened version of the Symptoms Checklist-90, questioning physical and psychological symptoms across nine dimensions: somatization; obsession-compulsion; interpersonal sensitivity; depression; anxiety; hostility; phobic anxiety; paranoid ideation and psychoticism, plus a global score (Global Severity Index). The BSI contains 53 items. Participants rate each item on a 5-point scale ranging from 0 (not at all) to 4 (extreme).

### Data analysis

Bassists can play their instrument either right- or left-handed. The movement patterns of the hand which sounds the strings (above the resonance box) via the fingers, a plectrum or bow are different from those of the hand (at the neck of the bass) responsible for the melody. For this reason, the terms ‘left’ and ‘right’ were substituted in the data analysis by ‘neck side’ and ‘box side’. The complaint scores related to the shoulder and forearm locations (due to elevated positioning of the arm in playing the double bass) were clustered as right and left ‘shoulder area’, respectively. Because all of the muscles inserted at the wrist originate from the forearm, complaint scores from the wrist and forearm (at the right side due to the flexed position of the wrist in playing the bass guitar) were clustered as right and left ‘wrist area’, respectively. The MSC scores were dichotomized into ‘no complaints’ (answering categories ‘no complaints’ or ‘rarely’ for the body region) and ‘complaints’ (‘often’ or ‘always’). Since the shoulder and wrist areas consisted of several separately scored body regions, the highest scores for frequency of complaints and pain intensity were taken as the score for that area.

The health-related items of smoking, alcohol use, drug abuse and body mass index (BMI) were dichotomized into an ‘objective health score’ of ‘healthy’ versus ‘unhealthy’. ‘Unhealthy’ was scored when at least one of the following was present: smoking more than 21 cigarettes or consuming more than 21 units of alcohol a week, using hard drugs (yes) and/or a BMI score lower than 18 or higher than 25. The data from the question about ‘playing another instrument for at least 5 h a week’ were dichotomized into a score of ‘0’ if ‘no’ and ‘1’ if ‘yes’.

### Statistical analysis

Demographic and playing characteristics are presented as means and standard deviations (for interval/ratio data), medians (for ordinal data) and percentages (for nominal and dichotomized data). The interval/ratio data were tested for normal distribution (with the Shapiro–Wilk test because half of the subpopulations included <50 bassists).

As the first step in the analyses, we determined the differences in frequencies of MSC scores (during the last 3 months in the various joint areas of the upper body) between the multi- and mono-instrumentalists. Analyses were performed using the Chi-square test or Fisher’s exact test for dichotomous data. Fisher’s exact test was used instead of the Chi-square test if there was insufficient data in one or more cells. Body regions showing an association with MSC (or a tendency toward one) with *p* < 0.20 were selected for further analyses.

Secondly, relevant non-MSC variates were selected for the final step. Analyses were performed using the *t* test for normally distributed data, the Mann–Whitney *U* test for non-normally distributed data and the Chi-square test or Fisher’s exact test for dichotomous data, with the potential confounder as one variable and the MSC in the selected body areas as the dependent variable. The third and final step consisted of a backward stepwise logistic regression to ascertain the effects of multi/mono-instrumentalism and covariates on the likelihood that participants had MSC in the body areas found in step 1. Multi- or mono-instrumentalism, and the variables showing an association with a *p* value <0.20 in step 2, were entered as the independent variable, while MSC in the selected body area was entered as the dependent variable. Variables such as ‘playing both bass instruments’ and ‘playing another instrument’ are directly related to ‘multi-instrumentalism’, and ‘problems carrying equipment’ is a consequence rather than a covariate. Therefore, these variates were excluded from the final analysis.

Level of significance in the final models was set at *p* ≤ 0.05, two-tailed. All data were analysed using SPSS, version 20.

## Results

The study sample consisted of 141 bassists: 56 double bassists (39.7%), 41 bass guitarists (29.1%), 44 (31.2%) bassists playing both bass instruments and 35 bassists (24.8%) playing another instrument for at least 5 h a week. Of the multi-instrumentalists, 65% played both bass instruments and 51% played another instrument besides one or both types of bass instruments, indicating that at least 15% of the study population played ≥3 types of instruments (=two bass instruments and another instrument or one bass instrument and two or more other instruments; the number of multi-instrumentalists playing one type of bass instrument and at least two other instruments is not known). Detailed sample characteristics, the scores of the potential confounders and MSC characteristics are presented in Online Resource 2. A summary is presented in Table 1. The subgroups of bassists in this paper are different from those in our previous paper, despite the same study population because of a different definition of the subgroups in the hypothesis.

Nearly three quarters of the bassists (73.8%) reported MSC. In step 1 of the analyses, only MSC of the left shoulder area showed an association (or a tendency towards one) with multi-instrumentalism, with a *p* value <0.20 (*p* = 0.025) (see Table 2).

**Table 2:** Association between MSC body areas and mono- versus multi-instrumentalism

	Mono- versus multi-instrumentalism			*P*
	Mono-instrumentalism	Multi-instrumentalism	Total	
	Column *N* (%)	Column *N* (%)	Column *N* (%)	
Neck complaints (in last 3 months)				
Always and often	25 (34.2)	17 (25.0)	42 (29.8)	0.271
Back complaints (in last 3 months)				
Always and often	27 (37.0)	32 (47.1)	59 (41.8)	0.237
Left shoulder area complaints (in last 3 months)				
Always and often	10 (13.7)	23 (33.8)	33 (23.4)	**0.025**
Right shoulder area complaints (in last 3 months)				
Always and often	17 (28.3)	15 (18.5)	32 (22.7)	0.222
Left wrist area complaints (in last 3 months)				
Always and often	15 (25.0)	19 (23.5)	34 (24.1)	0.845
Right wrist area complaints (in last 3 months)				
Always and often	13 (17.8)	14 (20.6)	27 (19.1)	0.831

*Shoulder area* shoulder and upper arm, *wrist area* wrist and lower arm, *Bold* variates *p* < 0.20, entered in the binary logistic regression analysis (see text)

Online Resource 3 presents the detailed sample characteristics and the scores of the potential confounders in this association (results of step 2). Most (85.8%) bassists with MSC of the left shoulder area experienced mild pain (Numeric Rating Scale ≤3) in the left shoulder area during the last reported week. The complaints hindered 42.5% of the total bassist population in their work to some degree (8.5 and 34.0% for ‘always or often’ and ‘never and rarely’, respectively). The prevalence of MSC (in the last three months) for the total group of bassists was 23.4% for the left shoulder area. The multi-instrumentalists had more MSC in the left shoulder than the mono-instrumentalists: 33.8 and 13.7%, respectively.

There was a tendency for the mono-instrumentalists to play more hours than the multi-instrumentalists; playing less in the three lowest categories of hours/week (44 versus 59%), but more in the highest category of ≥22 h/week (56 versus 41%) (Table 1).

Because half of the multi-instrumentalists (51%) indicated that they played at least one other instrument beside the bass instrument(s), extra subgroup analyses were performed to control for the possible bias resulting from the fact that the extra playing hours on those instruments were not taken into account in the main analysis. The subgroup analyses (the differences in MSC of the upper body part and playing time between bassists playing both bass instruments without another instrument and mono-instrumentalists (*n*-106), in steps 1 and 2 of the analyses, respectively) yielded outcomes comparable to those of the main analyses (MSC of left shoulder area: *p* = 0.011; other body regions: *p* ≥ 0.20; playing time: *p* = 0.54). In view of these results, we performed the final step 3 only for the total group of bassists.

In the group of mono-instrumentalists, there were more double bass players (60%) than bass guitarists (40%). Since there was no difference in the prevalence of MSC in the left shoulder area (or the right wrist area) between these two types of bass instrumentalists (Woldendorp et al. 2015), no additional analyses were performed on this subgroup.

In the second step of the statistical analysis (Online resource 3) seven variables with *p* < 0.20 were identified (‘playing double bass’, ‘playing both bass instruments’, ‘playing classical music’, ‘playing Jazz’, ‘problems carrying equipment, ‘doing sports’, and ‘tinnitus’). As explained in the methods section, ‘playing both bass instruments’ and ‘problems carrying equipment’ were not entered in the final analysis. Thus, the variate ‘multi-instrumentalism’ and five covariates were entered in the third analysis. Three of these variables (i.e. multi-instrumentalism, playing classical music and doing sports) remained after elimination in the backward stepwise method (Table 3).

**Table 3:** Final step of the logistic regression analysis testing the hypothesis that bassists playing two or more types of instrument (multi-instrumentalists) would have the same prevalence of MSC in the left shoulder area as bassists playing one type of instrument (mono-instrumentalists)

	Unstandardized coefficient		Odds ratio	95% CI interval		*p* value
	B	Standard error		Lower	Upper	
Multi-instrumentalists (1) versus mono-instrumentalists (0)	−1.207	0.471	0.299	0.119	0.753	0.010
Playing classical music	−1.111	0.464	0.329	0.133	0.817	0.017
Performing sports	−1.187	0.461	0.305	0.124	0.753	0.010

Overall prediction percentage: 78.7%; Nagelkerke *R*^2^ 0.189

The test of our null hypothesis showed that there was actually a significant difference between the multi-instrumentalists and the mono-instrumentalists in the prevalence of MSC in the shoulder area at the neck side (mainly left). The odds ratio of the association between having MSC in the left shoulder area and mono/multi-instrumentalism was 0.30 (95% CI 0.11–0.753).

Two covariates, viz. ‘performing sports’ (yes = 1) and ‘playing classical music’ (yes = 0) were identified in the final model of logistic regression (Table 3) as also being associated with MSC. This indicates that musicians who performed sports were more likely to have MSC, and those who played classical music were less likely to have MSC in the left shoulder area.

## Discussion

The null hypothesis, i.e. that there was no difference in the prevalence of MSC in the majority of the body parts between mono- and multi-instrumentalists, was not rejected. Contrary to our expectation, multi-instrumentalism offered no protection against MSC compared to mono-instrumentalism. Our data suggest that variation in occupational load has no protective effect regarding MSC. Moreover, the odds ratio of the association between having MSC in the left shoulder area and mono/multi-instrumentalism was 0.30 (0.119–0.753; *p* = 0.019), indicating that multi-instrumentalists were approximately 3.3 times more likely to have MCS in this joint area than mono-instrumentalists. This suggests that variation in occupational loads is associated with a higher rather than a lower likelihood of having MSC.

The results of the present and our previous study (Woldendorp et al. 2015) may fuel the debate in the literature on pain and occupational medicine, as other studies in the field have reported the opposite results (Nyman et al. 2007; Wu 2007; Ranelli 2011). The results of our previous study indicated that the ‘poor’ playing position of the left shoulder area in double bassists and the right wrist area of bass guitarists were not associated with a higher prevalence of MSC. The present study shows that variation in playing posture (as a result of multi-instrumentalism) is actually associated with a higher prevalence of MSC in the left shoulder area, and the present study did not find a protective effect on the other joint areas of the upper body either.

We compared the results of this study to those of other studies among bassists (Gilbert 2008; Meidell 2011) and other instrumentalists (Ranelli 2011). A detailed comparison could not satisfactorily explain the discrepancy in the findings. The reason why no other publications have reported a negative association between variation in occupational exposure and MSC in the left shoulder area remains unclear.

Because of the cross-sectional design of our study, no inferences about causality can be made. We have considered several possible explanations for the remarkable finding of a higher prevalence of MSC in the left shoulder area in multi-instrumentalists. First, the mono-instrumentalists spent more hours a week playing music than the multi-instrumentalists (Table 1), although the difference was not statistically significant. The extra analyses showed that there was no statistically significant difference in playing time between the two groups if the multi-instrumentalists playing another instrument were excluded, while the association in this subgroup between MSC in the left shoulder area and mono/multi-instrumentalism remained. Hence, it is not likely that major differences in playing time can explain the finding that multi-instrumentalists in this study had more MSC in the left shoulder. We were unable to find any information in the literature about an association between multi-instrumentalism versus mono-instrumentalism and MSC, so we cannot compare our results with those of other studies about this topic (e.g. in the recent reviews by Baadjou et al. 2016; Kok et al. 2016; Silva and Afreixo 2015).

Second, assuming that multi-instrumentalists strive for the same excellent level of professional performance on their main instrument, their shorter exposure time might be compensated by more intensive practice, which might cause extra stress on the musculoskeletal system, resulting in MSC.

Third, playing the second (or third) instrument for only about 1 h a day (and not as closely guided by a good teacher, as it is not the main instrument) might result in a less ergonomic playing technique than that used when playing an instrument for about 1300 h a year. Therefore, the second instrument can have a greater additional negative impact on the body in comparison with the main instrument. This negative effect could cancel out a potential positive effect of variation in occupational load and fewer hours of playing. Our data do not provide enough details to test these explanations, so this might be a subject for further investigation.

The fourth possible explanation is that different types of instrument require different neuro-physiological movement programs, which may interfere with each other. Performing instrumental music at a professional level requires motor functions with highly skilled sequential and serial movements of groups of muscles. Musicians undergo thorough training, over thousands of hours, to be able to perform these movements at an automatic level (Altenmüller et al. 2012). This requires both peripheral and central anatomical and physiological adaptations (Altenmüller et al. 2012; Enoka 2008; Karni et al. 1995; Klöppel 2000; Münte et al. 2002; Otten 2009; Wagner 2005). According to Schmidt’s Schema Theory (Schmidt, 2003) about motor control and motor learning, these rapid complex movements of related groups of motor actions (‘generalized motor programs’) are retrieved from memory and then adapted to the current situation. Playing different types of instruments might cause interference between these fine-tuned instrument-specific generalized motor programs, as has been found for the combination of playing the piano and the violin (Wagner 2005). It is unknown whether this interference is associated with MSC. This might indicate that there is an optimum for the degree of variation and intensity in one’s occupation; at an expert level of performance, too much or too little variation may not be beneficial to the worker.

Alternative explanations for the unexpected results of our study might be related to selection bias, operationalizations of the independent and dependent variables differing from those in other studies, or a type I error. Compared to other studies among bassists, our study sample was larger and our analyses were adjusted for potential confounders (Woldendorp et al. 2015). A type I error might have arisen if one or more confounders were missed in the study. Our assumption of a multi-causal bio-psycho-social aetiology of MSC induced us to include various biological and psychological factors (assessed using the BSI) in our analysis. Nevertheless, some biological, psychological and/or social confounders may have been missed. In our previous study (Woldendorp et al. 2015), a burdensome occupational load on the left shoulder area was not found to be a potential biological confounding factor. It is not clear if the combination of playing a bass instrument and one particular other type of instrument in the group of multi-instrumentalists can explain our unexpected study outcome. Further research is suggested to test if certain characteristics of the variation in occupational load are responsible for the association we found. Previous studies have reported a correlation between psychological functions (van der Windt et al. 2000; Miranda et al. 2005; Walker-Bone et al. 2004), level of stress (van der Windt et al. 2000; Miranda et al. 2005) and work situation (Sala et al. 2010), MSC in general, and MSC in the shoulder area in particular. To our surprise, none of the BSI items, including a wide range of psychological factors such as neuroticism and level of psychological distress, was found to be associated with MSC. No potential social confounders were included in our study, except indirectly via the factors of ‘music style’ (Sataloff et al. 2010; Spahn et al. 2011) and ‘lifestyle’ such as smoking, alcohol and drug use. For example, the subculture of bass guitarists might be a major confounding factor in the association between multi-instrumentalism and MSC. It would be interesting to focus future research more on the potential impact of ‘social aspects’ in the association between MSC and the variation in occupational load. As mentioned above, we can give no valid explanation for our findings. Further speculation about a possible explanation is beyond the scope of the present study.

We conclude that, in this sample of professional bass players, multi-instrumentalism was not associated with less MSC, but actually with an approximately 3.3 times higher prevalence of MSC of the left shoulder area. The usual assumption of a protective effect of variation in (‘poor’) postures, and therefore variation in occupational exposure, on the prevalence of MSC was not confirmed.

## Electronic supplementary material

Below is the link to the electronic supplementary material.

Online Resource 1. Questionnaire (DOCX 25 kb)

Online Resource 2. Demographic and clinical characteristics of the bass guitarists and double bass players playing one instrument (mono-instrumentalists), the bassists playing two or more types of instruments (multi-instrumentalists) and the total group (DOCX 31 kb)

Online resource 3. First step of the statistical analysis testing the distribution of the variables over the bassist groups with and without MSC in the left shoulder area (DOCX 35 kb)

## Footnotes

1 For reasons of readability ‘the wrist area related to the box side or bow side of the instrument’ and ‘the shoulder area related to the neck side of the instrument’ are referred to below as the ‘right wrist area’ and ‘left shoulder area’, respectively, because nearly all bassists play/bow right-handed.
