# Supplementary material for: Variation in occupational exposure associated with musculoskeletal complaints: a cross-sectional study among professional bassists
Source: Int Arch Occup Environ Health. 2017 Oct 20;91(2):215–23. doi: 10.1007/s00420-017-1264-5 (PMC5797213; doi:10.1007/s00420-017-1264-5)
Supplement: Supplementary file 1 — Online Resource 1. Questionnaire (DOCX 25 kb) [file 420_2017_1264_MOESM1_ESM.docx]

**Online Resource 1. Questionnaire.**

1. Name

2. Age

3. Sex

4. What instruments do you play?

5. At what age did you start playing your instrument(s)?

6. How many hours a week do you work (or study) as a bassist? (write down your answer in hours a week)

7. Do you play other instruments (for more than 5 hours a week) besides the double or electric bass?

8. What kind of instrument?

9. What music genre do you play?(multiple answers possible)

| Classical |
| --- |
| Jazz |
| Pop |

10. If you play the double bass, do you use the German or the French Bow?

| French bow |
| --- |
| German bow |
| Both |
| I don’t use a bow |
| I don´t play double bass |

11. Do you practice a sport?

| Yes |
| --- |
| No |

12. How many hours a week?

| 1 |
| --- |
| 2 |
| 3 |
| 4 |
| 5 |
| 6 |
| 7 |
| 8 |
| 9 |
| 10 |
| 11 |
| 12 |
| 13 |
| 14 |
| 15 |
| 16 |
| 17 |
| 18 |
| 19 |
| 20 |
| more than 20 hours a week |
| N |

13. Do you smoke?

| Yes |
| --- |
| No |
| N |

14. How many cigarettes a day?

| 0-5 |
| --- |
| 6-10 |
| 11-15 |
| 16-20 |
| 21-25 |
| 26-30 |
| 31-35 |
| 36-40 |
| more than 40 cigarettes a day |
| N |

15. Do you drink alcohol?

| Yes |
| --- |
| No |
| N |

16. How many units a week?

| 0-5 |
| --- |
| 6-10 |
| 11-15 |
| 16-20 |
| 21-25 |
| 26-30 |
| 31-35 |
| 36-40 |
| 41-45 |
| 46-50 |
| 51-55 |
| 56-60 |
| more than 60 units a week |

17. Do you frequently (more than once a week) use drugs?

| Yes |
| --- |
| No |

18. What kind of drugs?

| Soft drugs |
| --- |
| Hard drugs |

19. How would you describe your general state of health?

| Excellent |
| --- |
| Very good |
| Good |
| Moderate |
| Poor |

20. Did you have surgery in the past?

| Yes |
| --- |
| No |

21. What kind of surgery?

22. Have you been diagnosed with a medical condition in the past?

| Yes |
| --- |
| No |

23. What kind of medical condition?

24. What is your weight? (in kg)

25. What is your height? (in cm)

26. In the last three months, have you had any complaints in the following body parts: neck, right shoulder, left shoulder, right upper arm, left upper arm, right elbow, left elbow, right lower arm, left lower arm, right wrist, left wrist, fingers of right hand, fingers of left hand, back, hearing problems, skin disorders?

| Always |
| --- |
| Often |
| Rarely |
| Never |

27. In the past (before 2009) have you had any complaints of the following body parts: neck, right shoulder, left shoulder, right upper arm, left upper arm, right elbow, left elbow, right lower arm, left lower arm, right wrist, left wrist, fingers of right hand, fingers of left hand, back, hearing problems, skin disorders?

| Always |
| --- |
| Often |
| Rarely |
| Never |

28. If you have had back pain: can you specify where the pain is located

| I have no back pain |
| --- |
| Lower back right |
| Lower back left |
| Lower back both sides |
| Centre right |
| Centre left |
| Centre both sides |
| Upper back right |
| Upper back left |
| Upper back both sides |

29. Do you suffer from loss of muscle power in one of the following body parts: neck, right shoulder, left shoulder, right upper arm, left upper arm, right elbow, left elbow, right lower arm, left lower arm, right wrist, left wrist, fingers of right hand, fingers of left hand?

| Yes |
| --- |
| No |

30. Did the complaints started suddenly?

| Yes |
| --- |
| No |

31. Was there a specific trigger at the beginning?

| Yes |
| --- |
| No |

32. How severe was the pain in the last week? (Average)
0= no pain 10= worst pain: neck, right shoulder, left shoulder, right upper arm, left upper arm, right elbow, left elbow, right lower arm, left lower arm, right wrist, left wrist, fingers of right hand, fingers of left hand, back.

| 0 |
| --- |
| 1 |
| 2 |
| 3 |
| 4 |
| 5 |
| 6 |
| 7 |
| 8 |
| 9 |
| 10 |

33. How severe was the pain at the WORST moment in the last week?
0= no pain 10= worst pain: neck, right shoulder, left shoulder, right upper arm, left upper arm, right elbow, left elbow, right lower arm, left lower arm, right wrist, left wrist, fingers of right hand, fingers of left hand, back

| 0 |
| --- |
| 1 |
| 2 |
| 3 |
| 4 |
| 5 |
| 6 |
| 7 |
| 8 |
| 9 |
| 10 |

34. How severe was the pain at the BEST moment in the last week?
0= no pain 10= worst pain: neck, right shoulder, left shoulder, right upper arm, left upper arm, right elbow, left elbow, right lower arm, left lower arm, right wrist, left wrist, fingers of right hand, fingers of left hand, back

| 0 |
| --- |
| 1 |
| 2 |
| 3 |
| 4 |
| 5 |
| 6 |
| 7 |
| 8 |
| 9 |
| 10 |

35. Do the complaints worsen when you are playing music in your neck, right shoulder, left shoulder, right upper arm, left upper arm, right elbow, left elbow, right lower arm, left lower arm, right wrist, left wrist, fingers of right hand, fingers of left hand, back, hearing problems, skin disorders?

| Always |
| --- |
| Often |
| Rarely |
| Never |
| I have no complaints |

36. Is there a relationship between your complaints and the transport of equipment (amplifier, bass guitar, double bass)

| Yes |
| --- |
| No |

37. Do you have a numbness and/or tingling sensation in your fingers?

| Yes |
| --- |
| No |

38. Can you point out which finger(s)?

| Thumb |
| --- |
| Index finger |
| Middle finger |
| Ring finger |
| Little finger |

39. Which hand?

| Right hand |
| --- |
| Left hand |

40. Do you have a numbness and/or tingling sensation during the night?

| Yes |
| --- |
| No |

41. Headache

| I have no headaches at all |
| --- |
| I have slight headaches which come infrequently |
| I have moderate headaches which come infrequently |
| I have moderate headaches which come frequently |
| I have severe headaches which come frequently |
| I have headaches almost all the time |

42. Is there a link between the headache and other complaints?

| Yes |
| --- |
| No |
| Don’t know |

43. If yes, what complaints?

44. Do you have tinnitus? (a continual noise in the ear such as a ringing or
roaring)

| Yes |
| --- |
| No |

45. Do have any trouble following a conversation in a noisy room?

| Always |
| --- |
| Often |
| Rarely |
| Never |

46. Is your hearing impairment limiting your profession as a musician?

| Yes |
| --- |
| No |
| I don´t have a hearing impairment |

47. Because of my complaints (all the complaints, not only hearing impairments) I have difficulties performing activities of daily living (showering, cooking, dressing etc)

| Always |
| --- |
| Often |
| Rarely |
| Never |

48. Do your complaints impair your ability to work as a bass or double bass player?

| Always |
| --- |
| Often |
| Rarely |
| Never |

49. If you assign ten points to your working ability (as a bass player) in the best period of your life, how many points would you give it at the moment? (0= not able to work; 10= equal to best period)

| 0 |
| --- |
| 1 |
| 2 |
| 3 |
| 4 |
| 5 |
| 6 |
| 7 |
| 8 |
| 9 |
| 10 |

50. How much time per week do you spend at the computer? (please write down in hours a week)

51. Do you have another job besides your work as a bassist? (that is, work not involving bass playing)

| Yes |
| --- |
| No |

52. What kind of job?

53. In your other work, do you perform a lot of repetitive movements?

| Yes |
| --- |
| No |

**Items from the Brief Symptom Inventory:**

DURING THE PAST 7 DAYS, how much were you distressed by:

0 = Not at all; 1 = A little bit; 2 = Moderately; 3 = Quite a bit; 4 = Extremely; R = Refused

Nervousness or shakiness inside?

Faintness or dizziness?

The idea that someone else can control your thoughts?

Feeling others are to blame for most of your troubles?

Trouble remembering things?

Feeling easily annoyed or irritated?

Pains in the heart or chest?

Feeling afraid in open spaces?

Thoughts of ending your life?

Feeling that most people cannot be trusted?

Poor appetite?

Suddenly scared for no reason?

Temper outbursts that you could not control?

Feeling lonely even when you are with people?

Feeling blocked in getting things done?

Feeling lonely?

Feeling blue?

Feeling no interest in things?

Feeling fearful?

Your feelings being easily hurt?

Feeling that people are unfriendly or dislike you?

Feeling inferior to others?

Nausea or upset stomach?

Feeling that you are watched or talked about by others?

Trouble falling asleep?

Having to check and double check what you do?

Difficulty making decisions?

Feeling afraid to travel on buses, subways, or trains?

Trouble getting your breath?

Hot or cold spells?

Having to avoid certain things, places, or activities because they frighten you?

Your mind going blank?

Numbness or tingling in parts of your body?

The idea that you should be punished for your sins?

Feeling hopeless about the future?

Trouble concentrating?

Feeling weak in parts of your body?

Feeling tense or keyed up?

Thoughts of death or dying?

Having urges to beat, injure, or harm someone?

Having urges to break or smash things?

Feeling very self-conscious with others?

Feeling uneasy in a crowd?

Never feeling close to another person?

Spells of terror or panic?

Getting into frequent arguments?

Feeling nervous when you are left alone?

Others not giving you proper credit for your achievements?

Feeling so restless you couldn’t sit still?

Feelings of worthlessness?

Feeling that people will take advantage of you if you let them?

Feeling of guilt?

The idea that something is wrong with your mind?
