# Supplementary material for: Variation in occupational exposure associated with musculoskeletal complaints: a cross-sectional study among professional bassists
Source: Int Arch Occup Environ Health. 2017 Oct 20;91(2):215–23. doi: 10.1007/s00420-017-1264-5 (PMC5797213; doi:10.1007/s00420-017-1264-5)
Supplement: Supplementary file 2 — Online Resource 2. Demographic and clinical characteristics of the bass guitarists and double bass players playing one instrument (mono-instrumentalists), the bassists playing two or more types of instruments (multi-instrumentalists) and the total group (DOCX 31 kb) [file 420_2017_1264_MOESM2_ESM.docx]

|  | | Mono- versus Multi-instrumentalism | | | | | |  |
| --- | --- | --- | --- | --- | --- | --- | --- | --- |
|  |  | *Mono-instrumentalism* | | Multi-instrumentalism | | Total | | P |
|  |  | Mean | Column  N (%) | Mean | Column  N (%) | Mean | Column  N (%) |  |
| Age | years (SD) | 34.7 (14.2) |  | 35.3 (15.8) |  | 35.0 (15.0) |  | 0.58 |
| Gender | male |  | 63 (86.3) |  | 62 (91.2) |  | 125 (88.7) | 0.523 |
| Bass guitar | yes |  | 29 (39.7) |  | 12 (17.6) |  | 41(29.1) | 1.000 |
| Double bass | yes |  | 44 (60.3) |  | 12 (17.6) |  | 56 (39.7) | 0.140 |
| Both bass instruments | yes |  | 0 (0.0) |  | 44 (64.7) |  | 44 (31.2) | 0.123 |
| Multi-instrumentalism | yes |  | 0 (0.0) |  | 35 (51.5) |  | 35 (24.8) | 0.025 |
| Number of hours playing/week | < 8 hours a week |  | 4 (5.5) |  | 7 (10.3) |  | 11 (7.8) | 0.234 |
|  | >7 to <15 hours a week |  | 11 (15.1) |  | 11 (16.2) |  | 22 (15.6) |  |
|  | >14 to <22 hours a week |  | 17 (23.3) |  | 22 (32.4) |  | 39 (27.7) |  |
|  | > 21 hours a week |  | 41 (56.2) |  | 28 (41.2) |  | 69 (48.9) |  |
| Classical music | yes |  | 42 (57.5) |  | 50 (73.5) |  | 92 (65.2) | 0.055 |
| Jazz | yes |  | 61 (83.6) |  | 67 (98.5) |  | 128 (90.8) | 0.070 |
| Pop | yes |  | 61 (83.6) |  | 62 (91.2) |  | 123 (87.2) | 0.538 |
| If you play the double bass, do you use the German or the French Bow? | French bow |  | 22 (30.1) |  | 25 (36.8) |  | 47 (33.3) | 0.952 |
|  | German bow |  | 15 (20.5) |  | 19 (27.9) |  | 34 (24.1) |  |
|  | both |  | 3 (4.1) |  | 8 (11.8) |  | 11 (7.8) |  |
|  | no bow |  | 7 (9.6) |  | 8 (11.8) |  | 15 (10.6) |  |
|  | don't play double bass |  | 26 (35.6) |  | 8 (11.8) |  | 34 (24.1) |  |
| Do you practise a sport? | yes |  | 34 (46.6) |  | 34 (50.0) |  | 68(48.2) | 0.008 |
| Do you smoke? | yes |  | 21 (28.8) |  | 17 (25.0) |  | 38 (27.0) | 0.487 |
| Do you drink alcohol? | yes |  | 59 (80.8) |  | 51 (75.0) |  | 110 (78.0) | 0.808 |
| Do you frequently use drugs? | yes |  | 5 (6.8) |  | 6 (8.8) |  | 11 (7.8) | 0.701 |
| General state of health (subjective) | excellent, very good or good |  | 62 (84.5) |  | 56 (83.8) |  | 118 (84.4) | 0.729 |
| General state of health (Objective) | ‘Healthy’ if ‘0’ scores on smoking, alcohol, drugs and abnormal BMI |  | 62 (84.5) |  | 53 (77.6) |  | 115 (81.2) | 0.792 |
| Body Mass Index (BMI) | BMI <=18,5 of BMI >=30 |  | 5 (7.1) |  | 12 (17.9) |  | 17 (12.4) | 1.000 |
| Dominant playing hand | right hand is playing hand |  | 73 (100.0) |  | 67 (98.5) |  | 140 (99.3) | 1.000 |
| Neck complaints (in the last 3 months) | Always and often |  | 25 (34.2) |  | 17 (25.0) |  | 42 (29.8) | 0.271 |
| Back complaints (in the last 3 months) | Always and often |  | 27 (37.0) |  | 32 (47.1) |  | 59 (41.8) | 0.237 |
| Left shoulder area complaints  (in the last 3 months) | Always and often |  | 10 (13.7) |  | 23 (33.8) |  | 33 (23.4) | 0.006 |
| Right shoulder area complaints  (in the last 3 months) | Always and often |  | 17 (28.3) |  | 15 (18.5) |  | 32 (22.7) | 0.222 |
| Left wrist area complaints  (in the last 3 months) | Always and often |  | 15 (25.0) |  | 19 (23.5) |  | 34 (24.1) | 0.845 |
| Right wrist area complaints  (in the last 3 months) | Always and often |  | 13 (17.8) |  | 14 (20.6) |  | 27 (19.1) | 0.831 |
| Do you have tinnitus? | yes |  | 16 (21.9) |  | 18 (26.5) |  | 34 (24.1) | 0.152 |
| Hearing impairment? | yes |  | 2 (2.7) |  | 9 (13.2) |  | 11 (7.8) | 0.701 |
| Number of hours a week spent at computer | < 8 hours a week |  | 20 (27.4) |  | 13 (19.1) |  | 33 (23.4) | 0.498 |
|  | >7 to <15 hours a week |  | 21 (28.8) |  | 21 (30.9) |  | 42 (29.8) |  |
|  | >14 to <22 hours a week |  | 13 (17.8) |  | 18 (26.5) |  | 31 (22.0) |  |
|  | > 21 hours a week |  | 19 (26.0) |  | 16 (23.5) |  | 35 (24.8) |  |
| Is there a relationship between complaints and the transport of equipment (amplifier, bass guitar, double bass)? | yes |  | 35 (47.9) |  | 35 (51.5) |  | 70 (49.6) | 0.103 |
| Do your complaints impair your ability to work as a bass or double bass player? | yes |  | 4 (5.5) |  | 8 (11.8) |  | 12 (8.5) | 0.151 |
| Do you have another job besides  your work as a bassist? | yes |  | 44 (60.3) |  | 47 (69.1) |  | 91 (64.5) | 0.295 |
| In your other work, do you perform a lot of repetitive movements? | yes |  | 9 (31.0) |  | 8 (38.1) |  | 17 (34.0) | 0.763 |
| BSI somatization score items 2,7,23,29,30,33,37/ number of items | | 0.85 (0.58) |  | 0.92 (.61) |  | 0.88 (0.59) |  | 0.230 |
| BSI obs som items 5,15,26,27,32,36/ number of items | | 1.26 (0.77) |  | 1.33 (0.79) |  | 1.29 (0.78) |  |  |
| BSI interp.sens. som items 20,21,22,42/ number of items | | 0.98 (0.77) |  | 1.09 (0.92) |  | 1.03 (0.84) |  |  |
| BSI depr som items 9,16,17,18,35,50/ number of items | | 1.00 (0.73) |  | 1.02 (0.71) |  | 1.01 (0.72) |  |  |
| BSI anx som items 1,12,19,38,45,49/number of items | | 0.98 (0.65) |  | 1.03 (0.73) |  | 1.01 (0.69) |  |  |
| BSI host. som items 6,13,40,41,46/ number of items | | 0.94 (0.65) |  | 0.95 (0.62) |  | 0.94 (0.64) |  |  |
| BSI phob anx som items 8,28,31,43,47/ number of items | | 0.75 (0.59) |  | 0.77 (0.60) |  | 0.76 (0.59) |  |  |
| BSI paran idea som items 4,10,24,48,51/ number of items | | 0.92 (0.71) |  | 0.92 (0.75) |  | 0.92 (0.73) |  |  |
| BSI psych som items 3,14,34,44,53/ number of items | | 0.84 (0.61) |  | 0.91 (0.70) |  | 0.87 (0.65) |  |  |
| BSI score others | | 0.88 (0.63) |  | 0.98 (0.64) |  | 0.93 (0.64) |  |  |
| Global BSI (GSI) score/number of items | | 0.95 (0.58) |  | 0.90 (0.54) |  | 0.93 (0.56) |  |  |

Online resource 2: Demographic and clinical characteristics of the mono-instrumentalist and multi-instrumentalist bassist groups and the total group. Differences between the groups were tested with the t-test for normally distributed data, Mann Whitney U-test for non-normally distributed data, chi-square test for dichotomous data and Fisher’s exact test if data did not meet the criteria for Chi-square test. Health score ‘subjective’ = scored as healthy if indicated by the bassists themselves; Health score ‘objective’= scored as healthy if score is ‘0’ on four health-related categories (see text); BSI= Brief Symptom Checklist; shoulder area = shoulder and upper arm; wrist area = wrist and lower arm. Bold = covariates p <0.20, entered in the first step of the binary logistic regression analysis (see text).
