# Supplementary material for: Variation in occupational exposure associated with musculoskeletal complaints: a cross-sectional study among professional bassists
Source: Int Arch Occup Environ Health. 2017 Oct 20;91(2):215–23. doi: 10.1007/s00420-017-1264-5 (PMC5797213; doi:10.1007/s00420-017-1264-5)
Supplement: Supplementary file 3 — Online resource 3. First step of the statistical analysis testing the distribution of the variables over the bassist groups with and without MSC in the left shoulder area (DOCX 35 kb) [file 420_2017_1264_MOESM3_ESM.docx]

**Online resource 3: Characteristics of bassists with and without MSC in last 3 months in the left shoulder area.**

|  | | MSC left shoulder area | | | | | | |
| --- | --- | --- | --- | --- | --- | --- | --- | --- |
|  |  | Rarely and never | | Always and often | | Total | | P |
|  |  | Mean | Column  N (%) | Mean | Column  N (%) | Mean | Column  N (%) |  |
| Age | Years (SD.) | 35.5 (15.1) |  | 33.2 (14.4) |  | 35.0 (15.0) |  | 0.581 |
| Gender | male |  | 97 (87.4) |  | 28 (93.3) |  | 125 (88.7) | 0.791 |
| Bass guitar | yes |  | 32 (28.8) |  | 9 (30.0) |  | 41 (29.1) | 0.533 |
| Double bass | yes |  | 48 (43.2) |  | 8 (26.7) |  | 56 (39.7) | **0.074** |
| Both bass instruments | yes |  | 31 (27.9) |  | 13 (43.3) |  | 44 (31.2) | **0.083** |
| Multi-instrumentalism | yes |  | 48 (43.2) |  | 20 (66.7) |  | 35 (24.8) | **0.019** |
| Number of hours playing/week | < 8 hours a week |  | 6 (5.4) |  | 5 (16.7) |  | 11 ( 7.8) | 0.234 |
|  | >7 to <15 hours a week |  | 18 (16.2) |  | 4 (13.3) |  | 22 (15.6) |  |
|  | >14 to <22 hours a week |  | 32 (28.8) |  | 7 (23.3) |  | 39 (27.7) |  |
|  | > 21 hours a week |  | 55 (49.5) |  | 14 (46.7) |  | 69 (48.9) |  |
| Classical music | yes |  | 77 (69.4) |  | 15 (50.0) |  | 92 (65.2) | **0.041** |
| Jazz | yes |  | 98 (88.3) |  | 30 (100.0) |  | 128 (90.8) | **0.038** |
| Pop | yes |  | 98 (88.3) |  | 25 (83.3) |  | 123 (87.2) | 0.327 |
| If you play the double bass, do you use the German or the French Bow? | French bow |  | 37 (33.3) |  | 10 (33.3) |  | 47 (33.3) | 0.952 |
|  | German bow |  | 26 (23.4) |  | 8 (26.7) |  | 34 (24.1) |  |
|  | both |  | 9 (8.1) |  | 2 (6.7) |  | 11 97.8) |  |
|  | no bow |  | 11 (9.9) |  | 4 (13.3) |  | 15 (10.6) |  |
|  | don't play double bass |  | 28 (25.2) |  | 6 (20.0) |  | 34 (24.1) |  |
| Do you practise a sport? | yes |  | 47 (42.3) |  | 21 (70.0) |  | 68 (48.2) | **0.008** |
| Do you smoke? | yes |  | 32 (28.8) |  | 6 (20.0) |  | 38 (27.0) | 0.234 |
| Do you drink alcohol? | yes |  | 87 (78.4) |  | 23 (76.7) |  | 110 (78.0) | 0.508 |
| Do you frequently use drugs? | yes |  | 8 (7.2) |  | 3 (10.0) |  | 11 (7.8) | 0.426 |
| General state of health (subjective) | excellent, very good or good |  | 95 (85.6) |  | 23 (76.7) |  | 118 (84.4) | 0.729 |
| General state of health (Objective) | ‘Healthy’ if ‘0’ scores on smoking, alcohol, drugs and abnormal BMI |  | 89 (81.7) |  | 23 (79.3) |  | 26 (18.8) | 0.479 |
| Body Mass Index (BMI) | BMI <=18,5 of BMI >=30 |  | 14 (13.0) |  | 3 (10.3) |  | 17 (12.4) | 0.494 |
| Dominant playing hand | right hand is playing hand |  | 110 (99.1) |  | 30 (100.0) |  | 140 (99.3) | 0.787 |
| Is there a relationship between complaints and the transport of equipment (amplifier, bass guitar, double bass)? | yes |  | 51 (45.9) |  | 19 (63.3) |  | 70 (49.6) | **0.069** |
| Do you have tinnitus? | yes |  | 30 (27.0) |  | 4 (13.3) |  | 34 (24.1) | **0.102** |
| Hearing impairment? | yes |  | 8 (7.2) |  | 3 (10.0) |  | 11 (7.8) | 0.426 |
| Number of hours a week spent at a computer | < 8 hours a week |  | 26 (23.4) |  | 7 (23.3) |  | 33 (23.4) | 0.989 |
|  | >7 to <15 hours a week |  | 33 (29.7) |  | 9 (30.0) |  | 42 (29.8) |  |
|  | >14 to <22 hours a week |  | 25 (22.5) |  | 6 (20.0) |  | 31 (21.9) |  |
|  | > 21 hours a week |  | 27 (24.3) |  | 8 (26.7) |  | 35 (24.8)) |  |
| Do you have another job besides your work as a bassist? | yes |  | 74 (66.7) |  | 17 (56.7) |  | 91 (64.5) | 0.211 |
| In your other work, do you perform a lot of repetitive movements? | yes |  | 16 (36.4) |  | 5 (16.7) |  | 17 (34.0) | 0.324 |
| BSI somatization score items 2,7,23,29,30,33,37/ number of items | | 0.90 (0.57) |  | .82 (0.69) |  | 0.88 (0.59) |  | 0.230 |
| BSI obs som items 5,15,26,27,32,36/ number of items | | 1.30 (0.77) |  | 1.23 (0.83) |  | 1.29 (0.78) |  |  |
| BSI interp.sens. som items 20,21,22,42/ number of items | | 1.04 (0.81) |  | 1.01 (0.98) |  | 1.03 (0.78) |  |  |
| BSI depr som items 9,16,17,18,35,50/ number of items | | 1.02 (0.72) |  | 0.97 (0.73) |  | 1.01 (0.72) |  |  |
| BSI anx som items 1,12,19,38,45,49/number of items | | 1.01(0.66) |  | 1.02 (0.81) |  | 1.01 (0.69) |  |  |
| BSI host. som items 6,13,40,41,46/ number of items | | 0.97 (0.64) |  | 0.81 (0.63) |  | 0.94 (0.64) |  |  |
| BSI phob anx som items 8,28,31,43,47/ number of items | | 0.80 (0.60) |  | 0.59 (0.55) |  | 0.76( 0.59) |  |  |
| BSI paran idea som items 4,10,24,48,51/ number of items | | 0.96 (0.72) |  | 0.78 (0.75) |  | 0.92 (0.73) |  |  |
| BSI psych som items 3,14,34,44,53/ number of items | | 0.88 (0.66) |  | 0.83 (0.65) |  | 0.87 (0.65) |  |  |
| BSI score others | | 0.97 (0.60) |  | 0.79 (0.74) |  | 0.93 (0.64) |  |  |
| Global BSI (GSI) score/number of items | | 0.96 (0.56) |  | 0.79 (0.56) |  | 0.93 (0.56) |  |  |

Online resource 3: Demographic and clinical characteristics of the groups of bassists with and without MSC in the last 3 months in the left shoulder area.

Health score ‘subjective’ = scored as healthy if indicated by the bassists themselves; Health score ‘objective’= scored as healthy if score is ‘0’ on four health-related categories (see text); BSI= Brief Symptom Checklist; shoulder area = shoulder and upper arm; wrist area = wrist and lower arm. Bold = covariates p <0.20, entered in the first step of the binary logistic regression analysis (see text).
